# Two-Dimensional Material-Based Electrochemical Sensors/Biosensors for Food Safety and Biomolecular Detection

**DOI:** 10.3390/bios12050314

**Published:** 2022-05-09

**Authors:** Tao Li, Dawei Shang, Shouwu Gao, Bo Wang, Hao Kong, Guozheng Yang, Weidong Shu, Peilong Xu, Gang Wei

**Affiliations:** 1 College of Textile & Clothing, Qingdao University, No. 308 Ningxia Road, Qingdao 266071, China; lishilitao@qdu.edu.cn; 2 Qingdao Product Quality Testing Research Institute, No. 173 Shenzhen Road, Qingdao 266101, China; shangdawei@outlook.com; 3 State Key Laboratory, Qingdao University, No. 308 Ningxia Road, Qingdao 266071, China; qdgsw@126.com (S.G.); xpl@qdu.edu.cn (P.X.); 4 Qingdao Institute of Textile Fiber Inspection, No. 173 Shenzhen Road, Qingdao 266101, China; wangduanyangkingpo@outlook.com (B.W.); swdqd0532@outlook.com (W.S.); 5 College of Chemistry and Chemical Engineering, Qingdao University, No. 308 Ningxia Road, Qingdao 266071, China; konghao5@outlook.com (H.K.); yangguozheng123@outlook.com (G.Y.)

**Keywords:** two-dimensional materials, nanohybrids, electrochemical sensors, biosensors, food safety, biomolecular detection

## Abstract

Two-dimensional materials (2DMs) exhibited great potential for applications in materials science, energy storage, environmental science, biomedicine, sensors/biosensors, and others due to their unique physical, chemical, and biological properties. In this review, we present recent advances in the fabrication of 2DM-based electrochemical sensors and biosensors for applications in food safety and biomolecular detection that are related to human health. For this aim, firstly, we introduced the bottom-up and top-down synthesis methods of various 2DMs, such as graphene, transition metal oxides, transition metal dichalcogenides, MXenes, and several other graphene-like materials, and then we demonstrated the structure and surface chemistry of these 2DMs, which play a crucial role in the functionalization of 2DMs and subsequent composition with other nanoscale building blocks such as nanoparticles, biomolecules, and polymers. Then, the 2DM-based electrochemical sensors/biosensors for the detection of nitrite, heavy metal ions, antibiotics, and pesticides in foods and drinks are introduced. Meanwhile, the 2DM-based sensors for the determination and monitoring of key small molecules that are related to diseases and human health are presented and commented on. We believe that this review will be helpful for promoting 2DMs to construct novel electronic sensors and nanodevices for food safety and health monitoring.

## 1. Introduction

With the development of modern technology and society, people pay more and more attention to their own health and environmental protection issues. It is very important to detect and monitor pathogenic factors and environmental pollutants in real-time. Among the potential detection methods, electrochemical detection has attracted the interest of researchers. The core of electrochemical detection technology is an electrochemical sensor, in which the interaction between the substance to be tested and the sensitive nanomaterial produces electrochemically active substances, and the electrochemical sensor performs qualitative and quantitative analysis by converting these substances into electrical signals [1,2]. Electrochemical sensors have been widely used in food analysis, clinical detection, environmental monitoring, and many other fields due to their simple equipment, low cost, strong response signal, good selectivity, and high sensitivity [3,4,5].

According to different response signals, electrochemical sensors can be divided into the current-type, potential-type, and resistance-type sensors [6]. In addition, electrochemical sensors can be divided into semiconductor sensors, light sensors, thermal sensors, field-effect sensors, etc., according to the different detection signals of the converters. The different substances detected by sensors can be divided into gas sensors, ion sensors, biosensors, etc. Compared with traditional sensors, electrochemical sensors can sense materials without destroying the surrounding system, which has obvious advantages for real-time monitoring of analytes.

In recent years, nanomaterials have developed rapidly in many fields due to their excellent electrical, magnetic, thermal, and optical properties [7,8,9,10], especially in the field of electrochemical detection, which is often used in the conversion element or induction of electrochemical sensors to improve the sensitivity and detection limit of electrochemical sensors for target analytes [11,12,13]. Combining nanomaterials with electrochemical sensors and utilizing the characteristics of nanomaterials can improve the detection sensitivity of the sensors, shorten the detection time, and further stabilize the physical and chemical properties of the sensors, making the detection performance of the sensors improved significantly. Among those nanomaterials, two-dimensional materials (2DMs) have become a good choice for electrode materials and attracted widespread attention in the field of electrochemistry due to their high mechanical flexibility, large specific surface area, many active sites, good chemical stability, high electrical conductivity, and thermal conductivity [14,15,16]. Especially the excellent heterogeneous electron transfer ability, large specific surface area, and good adsorption capacity of 2DMs make them have great application potential in electrochemical sensing [17,18,19]. 

2DMs that are currently used to fabricate electrochemical sensors mainly include graphene and its chemical derivatives [20], transition metal dichalcogenides (TMDs) [21,22,23], transition metal–carbon/nitrides (MXenes), metal–organic frameworks (MOFs), and others [24,25]. However, the preparation of 2DMs still faces many difficulties and challenges; the lamellar structure is especially prone to aggregation, which affects the properties of the materials and limits their wide application. Therefore, rationally designing and developing high-performance 2DMs is highly necessary in order to improve their electrochemical activity and electrode surface electrochemical signals by element doping, functionalized compounding, surface modification, and sheet thickness control of 2DMs. Meanwhile, these efforts are helpful in improving the transmission efficiency and enhance the detection sensitivity and detection speed, showing increasing application potential in electrochemical sensors and biosensors.

In this article, we present recent advances in the preparation, functional modification, and electrochemical sensor applications of 2DMs. The current preparation methods of 2DMs are based on graphene, TMOs, TMDs, MXenes, and their composites, as well as their applications in electrochemical sensors and biosensors, especially in the detection of small biological molecules, antibiotics, and pesticides, are introduced systematically. Finally, the application prospects of 2DM-based electrochemical sensors in food analysis, clinical detection, environmental monitoring, and other fields are summarized. We believe that this work will be helpful for readers to understand the design and fabrication of various 2DM-based electrochemical sensors and biosensors, which will inspire the development of sensors/biosensors for high-performance monitoring of food and environmental safety.

## 2. Preparation of 2DMs

**Figure 1:**
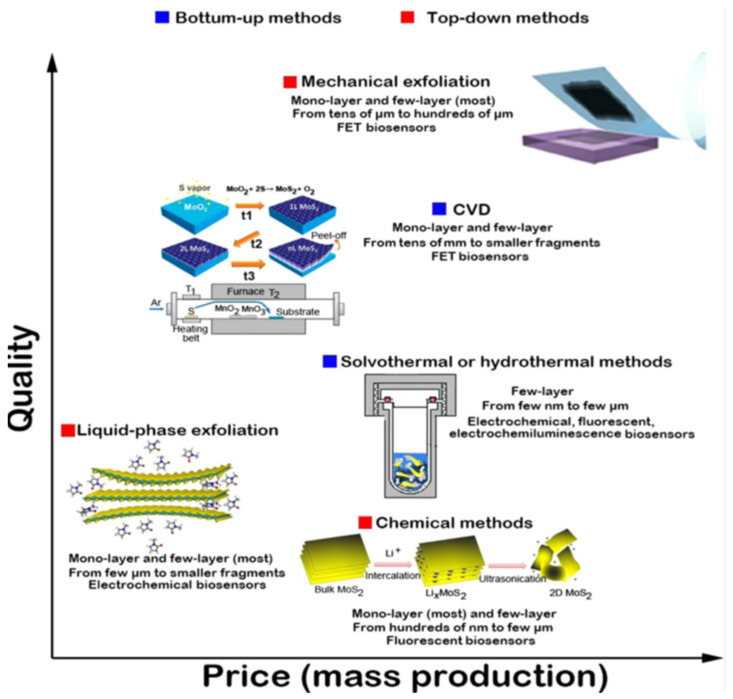
Several synthesis methods of mass-production of 2D MoS_2_, their characteristic size and thickness distributions of the resultant 2D MoS_2_, and correspondingly typical electrochemical biosensor application. Reprinted with permission from Ref. [26].

Different preparation methods greatly affect the properties of the obtained 2DMs, thereby affecting their applications in electrochemical sensing. In general, the preparation methods of 2DMs can be divided into two categories: Top-down methods and bottom-up methods, as shown in Figure 1 [26]. The top-down methods can usually prepare 2DMs by exfoliating layered bulk crystals into single-layer or multi-layer nanosheets by mechanical or chemical methods, mainly including mechanical exfoliation, chemical or electrochemical exfoliation, and liquid-phase exfoliation. The mechanical exfoliation method is derived from the “scotch tape method” of graphene, in which thin layers of graphene or TMD are exfoliated with tape and deposited onto the desired surface [27]. The thickness of 2DM nanosheets obtained by this method is difficult to control, and the yield is low, which limits the large-scale production of 2DMs. However, this method does not use any chemical substances, and the obtained materials maintain structural integrity and high crystallinity. In addition, the created 2DMs have good electronic and physical qualities, which are highly sensitive to environmental disturbances, and are very suitable as the candidates for the fabrication of electronic sensors [28,29,30]. For instance, Huang et al. [31] treated the substrate with oxygen plasma to remove surrounding adsorbates and introduced thermal treatment in the subsequent exfoliation process, which improved the mechanical exfoliation technique and increased the exfoliation rate and the area of the obtained graphene and Bi_2_Sr_2_CaCu_2_O_x_ nanosheets. This improved technique can be further used to produce other large-area 2DMs with improved yields.

Until now, liquid exfoliation has been shown to be an alternative method for large-scale production of 2DMs [32,33]. During this process, the layered bulk crystals undergo external perturbations (such as ultrasonic waves or mechanical shocks) in the solvent to expand the interlayer spacing, resulting in the final exfoliation of the material into ultrathin 2DMs. If the right solvent can be chosen, this approach works for any layered material held together by relatively weak Van der Waals bonds. The exfoliated nanosheets were stabilized against re-aggregation in a suitable solvent, whereas re-aggregation and precipitation occurred in an unsuitable solvent. In general, a suitable solvent must satisfy the following two conditions: first, it must be able to exfoliate the 2DMs at the highest concentration possible effectively; second, it can make the resulting 2DMs stable for the longest duration. For example, Zhang et al. [34] demonstrated that ultrasonic exfoliation of WS_2_, MoS_2_, and BN 2D layered nanomaterials in a mixed solution of ethanol and water and the obtained 2DM nanosheet suspension has high stability. This method is simple in process and low in cost, and it can be used to prepare ultra-thin 2DMs in large quantities, and additional dispersants or intercalating agents can be introduced into the system so that the functionalized 2DMs obtained in the exfoliation process can be obtained.

The chemical exfoliation method is usually based on ion intercalation (such as Li^+^), allowing other ions to enter the middle of the layered bulk crystal to expand the interlayer spacing, thereby weakening the Van der Waals interaction between adjacent layers of the bulk crystal, under mechanical vibration and ultrasonic treatment. Under the action of mechanical force, the bulky compound can be easily exfoliated into single-layer or multi-layer nanosheets. For example, Zhang et al. [14] developed an electrochemical Li^+^ intercalation-assisted liquid exfoliation method by adding layered bulk crystalline materials such as MoS_2_, WS_2_, TiS_2_, TaS_2_, ZrS_2_, or graphite as the cathode in the electrochemical cell. In this case, lithium foil was used as an anode, and during discharge, Li^+^ ions were driven into layered bulk crystals by electrochemical force and lithium foil to form lithium intercalation compounds. The coated electrodes covered with lithium intercalation compounds were subsequently removed, washed in water or ethanol, and sonicated to obtain nanosheet suspensions. After purification by centrifugation, single-layer or multi-layer nanosheets can be obtained with a high yield. This method easily leads to defects and structural deformation and is not suitable for studying the physicochemical properties of 2DMs; however, it is beneficial for the efficient production of monolayer MoS_2_ for the construction of fluorescent sensors and biosensors.

The top-down approach is only applicable to layered compound crystalline materials with limited use, while the bottom-up approach is more general, and all 2DMs can be prepared by this approach. The techniques for the bottom-up growth of 2DMs mainly include chemical vapor deposition (CVD) and solvothermal methods (also known as hydrothermal methods). The CVD technique means that the precursors containing the elements present in the 2DMs are thermally evaporated, introduced into the reaction chamber through the gas flow, and chemically reacted on the surface of a specific substrate to form the desired layered nanomaterials under appropriate reaction conditions [35,36,37]. For example, during the growth of MoS_2_ nanosheets, MoO_3_ and S powders are placed in a tube furnace with substrates such as SiO_2_ or sapphire and heated to about 800 °C. The vapor formed by the evaporated powder reacts on the surface of SiO_2_ to form layered MoS_2_, and the thickness of which depends on the growth parameters such as the flow rate of the carrier gas and the number of reactants [38]. Thus far, CVD has been successfully used to prepare many ultrathin 2DMs, including graphene, TMD (MoS_2_, WS_2_, MoSe_2_, Wse_2_, etc.), metal oxides (MOs), and others [39,40,41,42]. CVD technique enables the controlled growth of 2DMs with layer number, crystallinity, and lateral dimensions on different substrates with different precursors. However, the technology generally requires high temperature and high cost of inert gas and also requires specific substrates for transfer.

The solvothermal synthesis is a simple, low-cost, and high-yield method for the preparation of ultrathin 2DMs, which can be easily dispersed in organic or aqueous media for various applications [43]. Usually, the precursor solution is put into a high-pressure reactor, hydrothermally reacted under high temperature and high pressure, and then the nanomaterials are prepared by post-processing methods such as separation, washing, and drying. For example, Xie et al. [44] prepared defect-rich multilayer MoS_2_ nanosheets by the solvothermal method in the presence of excess thiourea. Dong et al. [45] prepared 2D Fe-doped NiO nanosheets with grain boundary defects using a solvothermal method through a thermally driven transformation process. The synthesized 2DMs were applied to electrocatalytic hydrogen evolution reactions. Won et al. [46] successfully synthesized a series of 2D SnS_x_Se_2−x_ nanosheets with a width of 0.20–2.00 μm and a thickness of 30–68 nm without topographical defects by a solvothermal reaction. The synthesized 2DMs have a band gap of 1.36–1.96 eV and exhibited promising applications in electronic, optoelectronic, and electrocatalytic aspects. Compared with CVD, the solvothermal method is easier to obtain much smaller crystals with high catalytic activity, which has great potential to construct electrochemical, fluorescence, and electrochemiluminescence sensors. The biggest challenge of this technology is how to block vertical growth while allowing lateral growth.

All of the above preparation methods have their own advantages and disadvantages. The choice of the preparation method depends on the specific application scenario of 2DMs. In different application systems, the factors that need to be considered include the cost, the amount of material required, desired level of crystal quality, biocompatibility, functional modification, and the final environment in which the 2DMs are used.

## 3. Structure and Surface Chemistry of 2DMs

**Figure 2:**
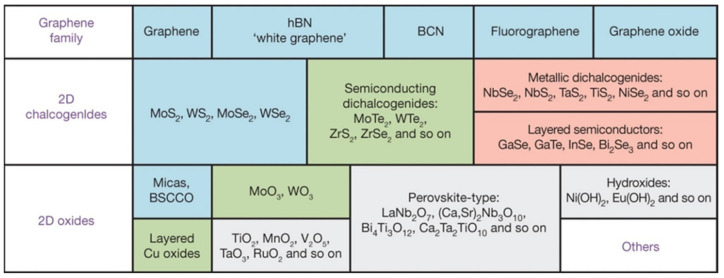
Current 2DMs. The color blue indicates the stable monolayers under ambient conditions, green indicates those probably stable in air, and pink indicates those unstable in the air but may be stable in an inert atmosphere. Grey indicates monolayers exfoliated by 3D compounds, and “Others” indicates 2D crystals, including borides, carbides, and nitrides [50].

Compared with zero-dimensional (0D), one-dimensional (1D), and three-dimensional (3D) nanomaterials, most 2DMs possess a larger specific surface area, which facilitates analyte loading and thus improves the sensitivity of material-based sensors. Moreover, the relatively large lateral dimensions of 2DMs can be in close contact with electrodes and thus, have attracted extensive attention in applications, including electrochemical sensors [47,48,49]. Currently known 2DMs mainly include graphene and its derivatives, transition metal dichalcogenides (TMDs) represented by MoS_2_, transition metal–carbon/nitrides (MXene), MOs, and others, as shown in Figure 2 [50]. 

### 3.1. Graphene 

Graphene is a honeycomb-like 2D structure composed of a single layer of sp^2^ carbon atoms, which is the basic building block of other carbon-based allotropes, such as graphite, carbon nanotubes, and fullerenes [51]. Graphene has many excellent properties, such as high transparency (97.7% visible light transmittance), high thermal conductivity (3 × 10^3^–5 × 10^3^ W m^−1^K^−1^), electrical conductivity (10^4^ Ω^−1^) at room temperature, high Young’s modulus (130.5 GPa), and high specific surface area (2630 m^2^g^−1^) [52,53,54]. In addition, graphene also has a special π-π* energy band structure, in which carbon atoms form a single-layer honeycomb lattice through sp^2^ hybridization, and its bandgap and conductivity are related to the number of layers and approach the bandgap and conductivity of graphite with the increase in the number of layers [55]. These unique structures provide it with excellent electrochemical performance while also showing excellent performance in many fields such as optical and biomedical disciplines.

Due to its large specific surface area, high electrical conductivity, abundant surface atoms, high mechanical strength, and potential for large-scale production, graphene has shown great potential in the field of electrochemical sensing [56,57]. First, graphene exhibits remarkably high carrier mobility and density at room temperature, which make it a good material for the construction of high-performance electrical devices [58,59]. Second, the graphene surface can interact with various analytes through Van der Waals forces, electron transfer, and covalent bonds, which lead to changes in graphene electronic properties, especially electrical conductivity [60,61]. The electron density inside graphene-based materials can be concentrated at its edges and structural defects, resulting in faster electron transfer rates than in the substrate plane, demonstrating the versatile properties of graphene as an electrocatalyst for electrochemical sensing applications [62]. Third, graphene has a high specific surface area, where each carbon atom is a surface atom, providing the largest surface area per unit volume, so charge transport through graphene is very sensitive to its chemical environment [63,64]. Fourth, graphene has excellent mechanical strength and flexibility, which are compatible with flexible wearable electronics [65,66]. The above-mentioned unique properties make graphene the most promising 2DM system in the field of electrochemical sensors.

Since the graphene surface plays a crucial role in intermolecular interactions, tuning the surface chemistry of graphene materials is the most important and direct way to tune their sensing performance. In order to enhance the interaction with the surrounding environment, the graphene surface is usually modified by various doping. The sensing performance in electrochemical sensing devices can be enhanced by incorporating functional groups or dopants after synthesis, as well as achieving structural and defect control and tuning the reactive sites of adsorbed chemicals. Compared with pristine graphene, graphene oxide (GO)-based gas sensors with defects and oxygen-containing functional groups have higher selectivity and sensitivity [67,68,69]. In addition, graphene materials can also be combined with other functional nanomaterials (such as metal nanoparticles [70], MO nanoparticles [71], conductive polymers [72], etc.) to form heterostructures, and the sensing properties can be further improved through the synergistic effect of different components. For example, the modification of graphene with conducting polymers can enhance the carrier scattering of graphene to form adsorbent layers that provide electrochemical interactions with a range of molecules [73], while the modification of enzymes can induce selectivity for biomolecules [74]. Combining graphene and MO for gas sensors can reduce the gas detection temperature and improve the sensitivity and response/recovery speed [75,76]. When applying graphene to electrochemical sensors, the effects of surface modification, lattice defects, and electronic properties on different surface chemical reactions should be considered.

### 3.2. TMDs

Two-dimensional TMDs have been favored by researchers due to their atomically thin structures and excellent electrical properties [77]. TMDs have a true 2D structure, but the physical properties are quite different from graphene. The monolithic form of TMDs has a layered structure of X-M-X, in which transition metal atoms (M) are sandwiched between two layers of chalcogen atoms, X, in a stoichiometric ratio of MX_2_. A common feature of these materials is that atoms in the same layer are covalently bonded into hexagons, and weak Van der Waals forces exist between adjacent layers, making stacking or thinning prone to occur [78,79]. TMDs can display a wide range of polymorphisms [80,81,82]. For example, MoS_2_ has four different crystal structures, namely 2H, 1T, 1T’, and 3R, depending on the coordination pattern between Mo and S atoms and the stacking order between layers [83]. The electrical conductivity of bulk TMDs also has various characteristics ranging from insulator (HfS_2_) to semiconductor (MoS_2_) and conductor (NbS_2_). The electronic properties of TMDs are significantly different from the bulk properties due to the fracture of the interlayer coupling [84,85]. When TMDs are exfoliated into 2D nanosheets, both basal planes and prism edges are exposed, exhibiting distinct structural and electronic properties. The basal surface of TMDs is mainly composed of chalcogenides, and on the edges of the prisms, metal or chalcogen atoms exist [41].

The advantages of TMDs, such as high thermal/chemical stability and abundant metal sites, make these 2DMs promising candidates for electrochemical sensing applications [86]. For example, the increased bandgap of MoS_2_ after exfoliation from bulk materials into nanosheets makes it an excellent choice for fabricating electronic devices [87]. The phase transition of MoS_2_ makes its transition from a semiconducting material to a metallic material, which enhances its electronic conductivity, and further optimizes its electrochemical properties [88,89]. In addition, the single-layer TMD nanosheets have a huge surface area, which enables their surfaces to adsorb biomolecules in large quantities. At the same time, 2D TMD nanosheets exhibit a very superior fluorescence quenching ability for fluorescent molecules, which also makes these materials have great potential in the construction of fluorescent biosensors [90,91].

**Figure 3:**
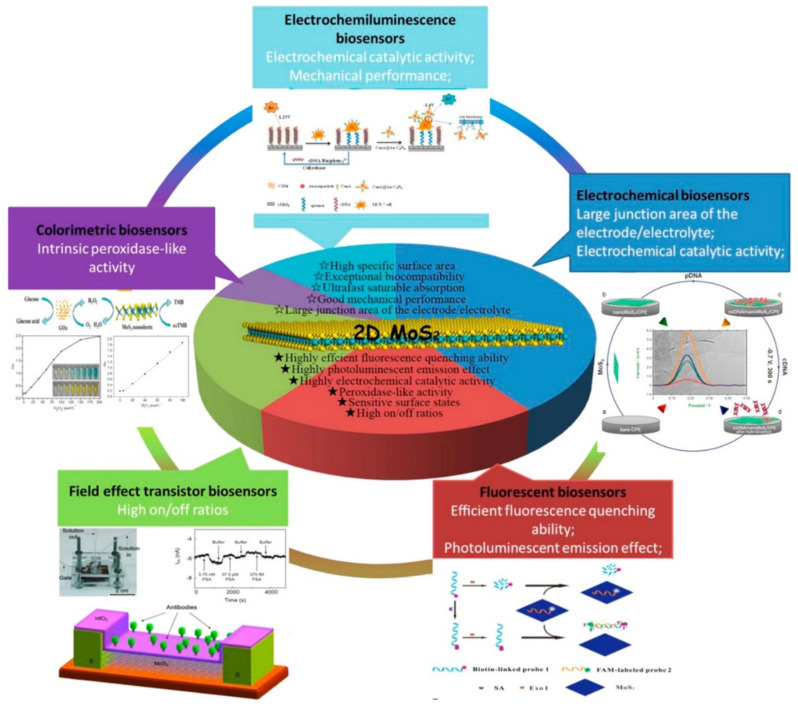
Intrinsic properties of 2D MoS_2_ and most representative properties applied in a specific biosensor. Reprinted with permission from Ref. [26].

The representative substances of TMDs include MoS_2_, SnS, and SnS_2_, among which MoS_2_ is the most widely studied one. Monolayer MoS_2_ is a semiconductor with a direct bandgap of 1.8 eV and a high absorption coefficient to generate efficient electron–hole pairs. This property largely compensates for the shortcomings of graphene (zero bandgaps), thereby broadening the application of 2DMs to ultrasensitive optoelectronic devices [92]. In addition, monolayer MoS_2_ has electron mobility >200 cm^2^V^−1^s^−1^ at room temperature, high current on/off ratio (1 × 10^8^), and high carrier lifetime (100 ps), which are suitable for use as a field-effect or electrochemical biosensor [93]. More importantly, the ultrathin planar structure of MoS_2_ confines the electrons/holes in the plane of atomic thickness, making it sensitive to the surrounding environment, and the edges can be terminated with Mo or S atoms by processing. This tunable electronic energy state makes it a promising substrate material for the construction of electrochemical sensors, offering great potential for various applications (Figure 3).

Theoretical calculation showed that MoS_2_ containing S vacancies had good adsorption capacity for several non-polar gases, while perfect MoS_2_ sheets had little or no adsorption for several non-polar gases [94]. The location and number of S vacancies at the edge of MoS_2_ nanosheets also have important effects on their stability and catalytic activity [95]. The S vacancies in MoS_2_ are most likely to form at the edges or corners of the nanosheets, and their specific locations/distributions depend on the size of the 2D nanosheets. Therefore, it is important to control the specific shapes of TMD nanosheets (hexagonal sheets, lamellar crystals, triangular sheets, etc.) in terms of morphology and size [96,97]. For small-sized MoS_2_ nanosheets, the shape can be engineered by the functionalization of the edge plane since the equilibrium geometry is highly sensitive to the energy of the edge atoms [98]. This molecular-level control enables great tunability of the electrochemical reactions and electronic properties of 2D TMD nanosheets, which can be applied in sensing technologies.

Although, MoS_2_ has many advantages, such as a suitable bandgap and large specific surface area, extraordinary biocompatibility, and easy functionalization. However, due to the dependence of Van der Waals interaction between the atomic layers of MoS_2_ [99], the conductivity tends to be low in the application process, and it is prone to agglomeration, which inhibits the electron transfer ability [100]. Therefore, it is necessary to compound some highly conductive materials (such as metal nanoparticles, carbon materials, conductive polymers, graphene, etc.) to solve this problem and make it better applied to sensor electrode materials [101,102,103]. The composition of 2D MoS_2_ with nanoparticles such as metals not only retains the advantages of large surface area and abundant active sites of 2D MoS_2_ but also improves the electrical conductivity. The synergistic effect produced by the combination of the two components can obtain nanocomposite materials with superior performance, which produces good results in electrochemical sensors. For example, Wang et al. [104] successfully combined 2D MoS_2_ and Au and used Ag nanospheres as a marker to detect carcinoembryonic antigen. Yagati et al. [105] prepared Au-MoS_2_ composites on indium tin oxide (ITO) substrates by electrodeposition and used them for the detection of T_3_ in clinical serum with good results. 

Since graphene has a similar microstructure and morphology to MoS_2_, it can be used as an ideal substrate for the growth of MoS_2_ nanosheets. The addition of graphene not only greatly improves the electrical conductivity but also promotes the formation of MoS_2_ nanosheets on graphene. The MoS_2_/graphene composites prepared by different methods have been shown to have good electrical conductivity and electrochemical performance in electrochemical sensors, which can measure antibiotics, ascorbic acid, dopamine, and uric acid [106,107,108].

### 3.3. TMOs

TMOs are compounds formed from metals and oxygen in the form of oxide ions and are the most diverse class of solids with various structures and properties. According to the existence of Van der Waals layered structure in the bulk, 2D TMOs can be divided into two categories: 2D layered TMOs (such as MoO_3_, TaO_3_, WO_3_, etc.) and 2D non-layered TMOs (ZnO, CuO, and SnO_2_ nanosheets or nanofilms, etc.) [48,109,110,111]. For example, orthorhombic MoO_3_ is a typical layered structure in which bilayer planar crystals of MoO_6_ octahedra are held together by Van der Waals forces in the vertical direction [112]. The weak interactions between these layers facilitate exfoliation by liquid or gas phase techniques to obtain nanosheets. Different TMOs have different electronic properties, and their electrical conductivities vary from metals to insulators. The conductivity of specific TMOs can be further tuned by changing crystal size, morphology, dopants, geometry, and temperature [113].

The ionic nature of oxygen ions and metal–oxygen bonds are two key factors that determine the surface properties of TMOs [114]. The unique properties of oxygen ions determine the performance of 2D TMOs in electrochemical sensings, such as molecular adsorption, charge transfer, and catalytic performance [115,116]. Specific energy states near or on the surface of 2D TMOs can generate Coulomb interactions with neighboring ions. Therefore, if the surfaces of two TMOs come into contact with each other, an interfacial potential is created, which changes the Fermi level of the surfaces [117]. Due to the strong ionic nature of TMOs, the surfaces of 2D TMOs can be electronically activated to enable various small molecules to adsorb on their surfaces. For example, at room temperature, oxygen is usually adsorbed on the surface of 2D TMOs in the form of negatively charged compounds [118]. The basal planes of layered MOs are terminated by oxygen atoms and have high chemical stability in air and water.

TMOs have been widely used in electrochemical sensors due to their semiconducting properties. At high temperatures (T > 250 °C), atmospheric oxygen increases the hole carrier concentration by binding to oxygen vacancies on the surface of MOs, enabling these materials to obtain stronger electrical conductivity at high temperatures [119,120]. However, the application of TMOs in sensing requires high operating temperatures, which consume a large amount of electricity relative to sensors that can operate at room temperature [121]. At the same time, due to the high working temperature, it has extremely high requirements for the environment and conditions of use, which greatly affects the service life and restricts the application range of TMOs sensors. In order to solve this problem, the sensing properties of materials are usually improved by doping or compounding with other semiconductor materials by using their synergistic effects [122,123,124]. For example, Xu et al. [125] used theoretical calculations to study the sensitivity of In-doped ZnO nanosheets to acetylene gas. It was found that the incorporation of In atoms into the ZnO nanosheets increased the adsorption energy of acetylene from −3.6 kcal/mol to −20.4 kcal/mol due to cationic interactions. In the presence of O_2_, H_2_O, N_2_, and CO_2_, the In-doped ZnO sheet can selectively detect acetylene gas with a short recovery time and high sensitivity. In another case, Zhou et al. [126] prepared NiO/ZnO nanosheets by a one-step hydrothermal method and examined their gas-sensing properties towards SO_2_. It was found that at the optimal operating temperature of 240 °C, the NiO/ZnO nanosheet-based sensor device had a response of 28.57 to 50 ppm SO_2_ and a gas detection range of 5–800 ppm.

### 3.4. Transition Metal–Carbons/Nitrides (MXene)

MXene is a new 2D transition metal–carbon/nitride with a structure similar to graphene [127,128,129]. In 2011, Naguib et al. [130] used concentrated hydrofluoric acid (HF) to selectively etch the Al atomic layer in the ternary layered cermet material Ti_3_AlC_2_ and successfully synthesized Ti_3_C_2_ nanosheets with layered morphology. A large number of functional groups, such as -OH, -F, and =O, are formed on the surface of MXene during the preparation process, giving it a unique structure, excellent electrical conductivity, and excellent properties such as environmental protection, which stimulates great enthusiasm for research and exploration [131]. 

**Figure 4:**
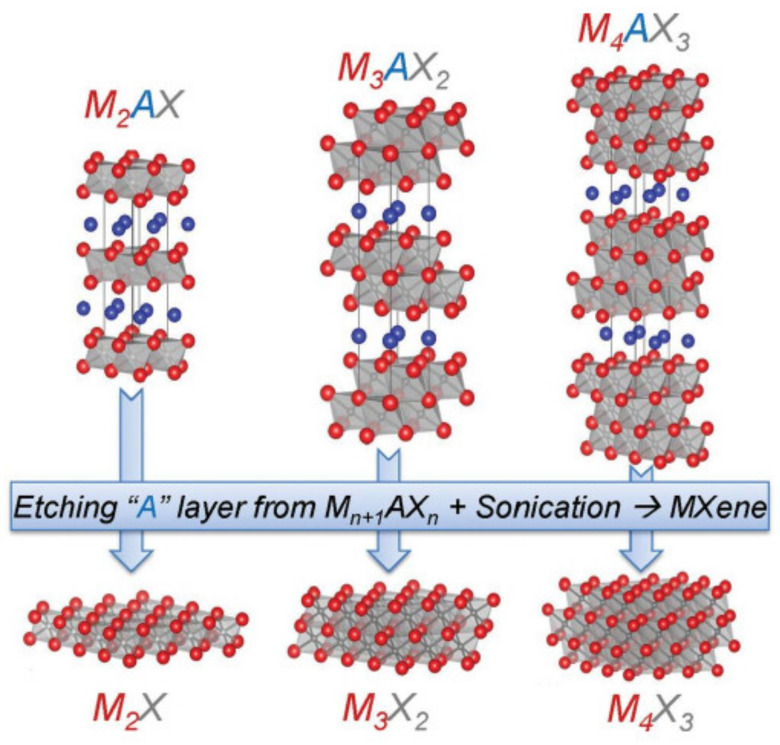
Schematic diagram of the structure of MXene and its corresponding MAX phase. Reprinted with permission from Ref. [135].

MXene is generally prepared by selective etching of its precursor MAX phase with concentrated hydrofluoric acid [132,133]. The MAX phase is a cermet material with a unique ternary layered structure, and its general formula is M_n+1_AX_n_, where M is a transition metal element, A is a group III or group IV main group element (mainly Al or Si), X is C and/or N elements, 1 ≤ *n* ≤ 3 [134]. In the MAX phase, there is a metallic bond between the M atom and A atom, while a mixed bond with a stronger binding force between the M atom and X atom mainly includes covalent, ionic, and metallic bonds. Therefore, it is possible to make use of the different bond energies between M-X and M-A to break the weaker M-A bond through some specific methods without destroying the M-X bond, thereby removing the A atomic layer and preparing a lamellar structure M_n+1_X_n_. According to the difference of n, there are mainly three structures 211, 312, and 413 (Figure 4) [135]. The MXene materials that have been successfully synthesized so far include Ti_3_C_2_, Ti_2_C, V_2_C, Ta_4_C_3_, (V_0.5_Cr_0.5_)_3_C_2_, and (Ti_0.5_Nb_0.5_)_2_C, etc. [136,137]. Among them, Ti_3_C_2_ is the most studied 2DM among MXene materials.

The surface activity of MXene prepared by the liquid etching method is extremely high, and it can rapidly react with water, fluoride ion, and oxygen in the solution to reduce the energy of the whole system. Therefore, MXene surfaces generally contain functional groups such as -OH, -F, and =O, and these functional groups are generally located in the most stable symmetrical positions in the MXene lattice structure [138,139]. By changing the surface functional groups, MXene materials can be switched between conductors and semiconductors. Theoretical calculations showed that after etching the A atomic layer in the MAX phase, the d-orbital electrons of the M atoms are rearranged, resulting in the metallic conductivity of the MXene nanosheet materials [140]. When the surface of the MXene nanosheet is occupied by functional groups such as -OH and -F, its band structure again exhibits semiconducting properties [130]. Mauchamp et al. [141] used high-resolution electron energy loss spectroscopy to study the properties of MXene materials and found that the surface plasmons could be tuned in the mid-infrared band of 0.2~0.7 eV by controlling the surface functional groups and thickness of MXene materials. In another study, Pellegrini et al. [142] found that plasmons can be used to enhance the performance of mid-infrared light sources, sensors, and detectors for chemical sensing and thermal imaging.

As 2D nanosheet materials, Mxene materials have more abundant elemental composition and complex mixed bonds than graphene materials. Therefore, MXene materials have more potential for modification towards specific applications. In order to improve the properties of MXene materials, other nanomaterials are combined with MXene to improve their original properties through synergic effects, thereby greatly improving the selectivity and sensitivity of MXene-based electrochemical sensors [143,144]. For example, Wang et al. [145] prepared TiO_2_-Ti_3_C_2_ nanocomposites based on Ti_3_C_2_ and used the created materials to immobilize hemoglobin (Hb) to construct a mediator-free hydrogen peroxide (H_2_O_2_) sensor with high stability and low detection limit. The experimental results showed that the TiO_2_-Ti_3_C_2_ nanocomposites had good biocompatibility with Hb and provided excellent protein biological activity and stability. The detection range was 0.1~380 μM with a detection limit of 14 nM. Meanwhile, the fabricated sensor could maintain 94.6% of the performance after 60 days.

### 3.5. Other 2DMs (BPs, C_3_N_4_, MOFs, 2D Polymers, and Others)

**Figure 5:**
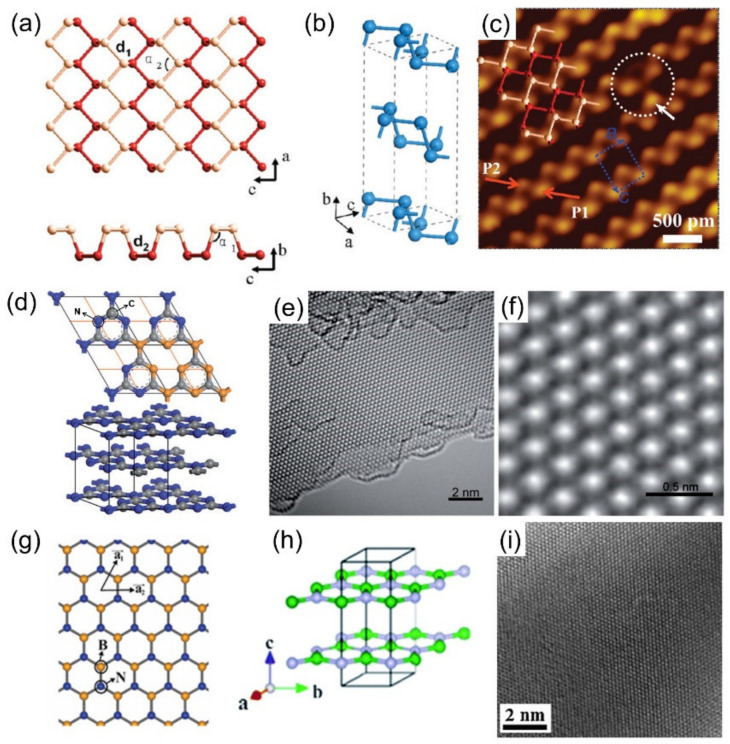
(**a**) Scheme of a single layer BP on the a-c and b-c plane. The upper and lower atoms are presented in pink and red, respectively. (**b**) Schematic illustration of unit cell. (**c**) High-resolution image of BP [148]. (**d**) Crystallographic unit cell and AB stacking arrangement of triazine-based g-CN layers. (**e**,**f**) HRTEM image of mechanically cleaved nanosheet of g-CN [158]. (**g**,**h**) Structure of h-BN, (**g**) The 2D floor plan; (**h**) Stereogram [164]; (**i**) HRTEM image of BN nanosheets [165].

Black phosphorus (BP), as an emerging 2D material, represents high carrier mobility and a tunable mild direct bandgap, which can be applied in the fields of electronics and optoelectronics. Two-dimensional BP is a single-layered crystal material composed of sp^3^ hybridized phosphorus atoms arranged in a layered orthorhombic structure with the space group [146]. BP has a distinctly nonplanar honeycomb lattice structure with lattice constants a = 3.31, b = 10.50, c = 4.38 Å [147]. BP also shows a folded conformation along with the armchair orientation, with the bilayer structure along the zigzag orientation predominant (Figure 5a) [148]. The folded structure creates unique intrinsic anisotropy, resulting in excellent exploitable electronic, optical, thermal, and mechanical properties. In addition, the biocompatibility of BP is very beneficial for biomedical applications. Similar to graphene, BP single crystals are bound by the Van der Waals forces and consist of vertically stacked and weakly crystalline layers with a distance of 5.5 Å from adjacent layers (Figure 5b). In each phosphorus layer, the P atoms exhibit a triangular pyramid structure due to the covalent bonding of phosphorus atoms with one lone electron pair, and defects may exist in the monolayer due to the loss of P atoms (Figure 5c), which favors the dissociation of the thin layer of black phosphorus atoms [148]. The mass production of BP thin layers is still in its infancy, and mechanical and liquid phase exfoliation are one of the most effective methods to obtain high-quality flake BP [149,150]. For example. Castellanos-Gomez et al. [151] used viscoelastic polydimethylsiloxane (PDMS) to exfoliate BP flakes after tape peeling, optimizing the peeling process, increasing the yield, and reducing the tape contamination. Brent et al. [150] performed liquid-phase exfoliation of BP in N-methylpyrrolidone (NMP), sonicated and centrifuged to obtain 3~5 layers of high-purity BP nanosheets (200 × 200 nm^2^). Zhao et al. [152] replaced traditional organic solvents with eco-friendly ionic liquids to prepare BP nanosheets, and the high-concentrated dispersions (0.95 mg mL^−1^) were stable in the environment for more than a month. 

The rapid development of BP-based gas/small molecule sensors has stimulated applications in biomolecule detection due to their superior sensing performance [153]. For instance, Chen et al. [154] prepared a BP-based FET biosensor for sensitive detection of antigen–antibody interactions. The mechanically exfoliated layered BP was passivated by Al_2_O_3_ to form the sensing/conducting channel of the FET, which was subsequently further modified with antibody-conjugated AuNPs. The antigen bound to the black phosphorus surface generated a negative gate potential, which changed the conductivity of the biosensor. The fabricated FET sensor could be used as a stable and rapid immunoglobulin G (IgG) diagnostic tool with a detection limit of 10 ng mL^−1^.

g-C_3_N_4_, a similar graphite material, is a layered structure composed of interlayer Van der Waals force [155,156,157]. The crystal structure of g-C_3_N_4_ is generally considered to be an N-substituted graphitic phase framework structure (Figure 5d–f), which is formed by sp^2^ hybridization of C and N atoms [158]. Bulk C_3_N_4_ can be processed by exfoliation and other methods to obtain nanoscale g-C_3_N_4_, such as g-C_3_N_4_ nanosheets, whose properties are very different from those of bulk materials. g-C_3_N_4_ nanosheets have good thermal and chemical stability, fluorescence effect, and other excellent properties [159,160,161]. g-C_3_N_4_ contains a small amount of hydrogen in the form of primary and/or secondary amine groups at its terminal due to incomplete condensation during the synthesis. The presence of terminal hydrogens and the high electron affinity of nitrogen for many analytes result in g-C_3_N_4_ with rich surface properties, including basic surface functions, electron-rich properties, and H-bond motifs [162]. The C and N elements of the g-C_3_N_4_ nanosheets are uniformly dispersed in each nanosheet with good thermal stability and tunable bandgap. Its excellent properties make it widely used in photocatalysis, photovoltaic power generation, bioimaging, and sensing [161].

Hexagonal boron nitride (h-BN) is another graphitic analog that has a periodic structure similar to graphene in plane, but with a different stacking order [163,164]. Adjacent layers with a distance of 3.30–3.33 Å gather together under the Van der Waals force to form bulk crystals (Figure 5g–i) [164,165]. h-BN is an electrical insulator with a bandgap of 5.2 eV, high thermal conductivity, excellent dielectric properties, and high-temperature oxidation resistance [166,167]. Within the BN nanosheets, the chemical alternation of boron (B) and nitrogen (N) atoms results in the ionic nature of this crystal, which can be highly sensitive to various gases. The adsorption of gas molecules greatly changes the electronic properties of BN nanosheets, which is a prerequisite for the fabrication of gas sensors. In addition, 2D BN nanosheets allow all of their atoms to be exposed to adsorbed gas molecules, thereby increasing the sensitivity of the sensors. Due to its high thermal stability and chemical inertness, BN nanosheets can also be used in harsh environments where other materials cannot. According to theoretical calculations, doped or defective h-BN nanosheets can respond to several gas molecules. For example, Ma et al. [168] studied the Pd-doping behavior on the h-BN monolayer as well as related adsorption and sensing performance upon three SF_6_ decomposed species to explore its sensing potential. They found that the adsorption performance of Pd–BN monolayer upon SF6 decomposed species is in order as SOF_2_  >  SO_2_  >  SO_2_F_2_. After gas adsorption, the bandgap of the Pd–BN monolayer is remarkably changed; the sensitivity is obtained as −41.04%,  −108.14%, and 2.55% for SO_2_, SOF_2,_ and SO_2_F_2_ systems, respectively, which implies the superior sensing behavior upon SOF_2_ at room temperature. Yamini et al. [169] investigated the electronic response of pristine and topologically defected h-BN nanosheets toward NO molecule using density functional theory calculations and found that the HOMO/LUMO gap of the pristine and Stone–Wales defected h-BN sheet is significantly decreased about 42% and 35%, respectively, upon NO adsorption which may increase the electrical conductance of the sheet and it might be potentially used in NO sensors. In addition, BC_3_ nanosheets such as BN and graphene were also demonstrated to be used to fabricate electrochemical sensors as a suitable choice for Megazol detection [170].

## 4. Applications of 2DM-Based Electrochemical Sensors and Biosensors

### 4.1. 2DM-Based Electrochemical Sensors/Biosensors for Food Safety

#### 4.1.1. Detection of Nitrite

In food safety and quality analysis, electrochemical sensors have been widely used in the detection of pesticide residues, heavy metal ions, pathogenic bacteria, and toxins. The application of a large number of inorganic fertilizers in crop cultivation greatly increases the content of nitrite in the environment. Nitrite can inhibit the growth of microorganisms in the food industry and is often used as a dye for meat products. Therefore, nitrite is very likely to enter the human body, making hemoglobin irreversibly converted into methemoglobin, which cannot exchange oxygen normally, disrupt the oxygen delivery system, and cause hypoxia. It may also lead to the production of carcinogen N-nitrosamine in the stomach. The content of nitrite in drinking water should be less than 3 mg/L [171]. Therefore, a sensitive, rapid and accurate method for the determination of nitrite is necessary. A variety of methods were used to detect the content of nitrite effectively. Compared with other methods, electrochemical sensors have attracted the attention of researchers due to their advantages, such as fast detection speed, wide linear detection range, low detection limit, and high stability [172]. 2DMs, such as MoS_2_, graphene, and others, were widely used in the field of electrochemical detection because of their abundant active sites and large specific surface area [173,174,175].

**Figure 6:**
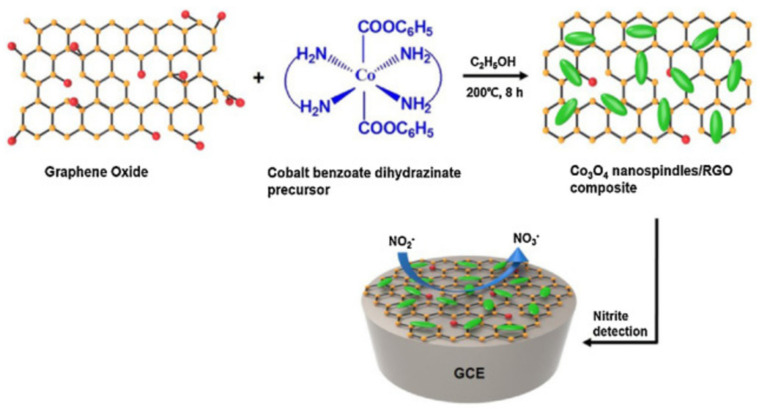
Schematic presentation on the fabrication of rGO and Co_3_O_4_-based electrochemical nitrite sensor. Reprinted with permission from Ref. [178].

However, the electrical conductivity of 2D MoS_2_ limits its application in the field of electrochemical detection. Compounding it with metal nanoparticles can effectively improve its detection sensitivity. For example, Zhang et al. [176] prepared 2D MoS_2_ by hydrothermal method and AuNPs by chemical reduction method. MoS_2_/AuNPs composites were prepared by mixing the as-prepared two components and further utilized for the fabrication of electrochemical sensors. By electrochemical tests, nitrite in tap water was detected. The MoS_2_/AuNPs composites-based electrochemical sensor exhibited a wide linear detection range (0.005–27.78 mM), low detection limit (1.7 μM), and good recovery rate, and the detection process is actually an oxidation process from nitrite to nitrate. In another study, Yang et al. [177] prepared 2D MoS_2_ by mechanical milling and ultrasonic liquid phase peeling. MoS_2_/Ni nanocomposites were prepared in situ by chemical reduction method and applied to electrochemical detection of nitrite with a wide detection range (5–800 μM) and low detection limit (2.48 μM). In addition, MoS_2_/Ni-modified electrode also has excellent detection characteristics such as high stability, repeatability, anti-interference, and recovery. Haldorai et al. [178] prepared electrochemical nitrite sensors based on spindle-shaped Co_3_O_4_ and rGO nanocomposites with good reproducibility, high stability, high sensitivity, and high selectivity in the process of nitrite detection, as shown in Figure 6. The fabricated sensor exhibited a detection limit of 0.14 μM with a linear detection range of 1–380 μM. Zhang et al. [179] prepared the Fe_3_O_4_/MoS_2_ nanocomposite structure of Fe_3_O_4_ nanospheres uniformly dispersed on MoS_2_ nanosheets by hydrothermal method. Fe_3_O_4_/MoS_2_ nanocomposites were used to modify the glassy carbon electrode (GCE) for the detection of nitrite with a wide detection range (1.0–2630 μM) and a low detection limit (0.5 μM).

#### 4.1.2. Detection of Heavy Metal Ions

Heavy metal ions (such as Pb^2+^, As^3+^, Cd^2+^, Hg^2+^, etc.) seriously harm the ecological environment and enter the animals and plants within the food chain, and eventually endanger human health [180,181]. Therefore, it is very important to develop a highly sensitive and selective method for the detection of heavy metal ions. In recent years, the electrochemical sensor has been used in the detection of heavy metals because of its low cost, fast response time, and easy operation. 

For example, Zhou et al. [182] used L-cysteine-functional graphene to fabricate an electrochemical sensor, which could simultaneously detect trace heavy metal Pb^2+^ and Cd^2+^ ions in food. Zhu et al. [183] carried out alkali intercalation treatment on the synthesized Ti_3_C_2_. The detection performance of Ti_3_C_2_ for heavy metal ions was studied by electrochemical analysis. The experimental results indicated that alkaline Ti_3_C_2_ has high sensitivity and a good linear relationship for the detection of heavy metal ions, and the detection limit is up to trace level. 

Graphene materials are very good candidates for the fabrication of electrochemical sensors for food safety. Mahmoudian et al. [184] constructed the electrode modified by Fe_3_O_4_ and rGO nanocomposites. The current density on the surface of the electrode is high (~400 μ A/cm^2^), and heavy metal ions such as Ag^+^, Cu^2+^, Hg^2+^, Bi^3+^, Cr^2+^, Fe^3+^, and other heavy metal ions did not interfere with the quantitative detection of Pb^2+^. Tan et al. modified single-stranded DNA aptamers on rGO by linear sweep voltammetry to construct a highly sensitive Hg^2+^ sensor, which could eliminate the interference of other metal ions and detect Hg^2+^ ions of 0.5 nM with high selectivity so as to detect the content of Hg^2+^ ions in water conveniently, quickly, and selectively [185]. Rahman et al. [186] prepared GO and silver nanowires (Ag NWs) composite-modified Pt electrodes for the determination of Hg^2+^. The synergistic effect of GO and conductive Ag NWs greatly promoted the electron transport and sensing ability of Hg^2+^. The detection limit of Hg^2+^ was 0.1 nM, which is significantly lower than the safety limit set by the World Health Organization. The sensor was used for the detection of Hg^2+^ in tap water samples with excellent performance, so it could be a promising field monitoring platform for Hg^2+^ in water. Wei et al. [187] prepared SnO_2_-rGO nanocomposites with uniform particle size and controllable structure under a one-step wet chemical method. Additionally, SnO_2_-rGO nanocomposites were used to modify the electrode to construct electrochemical sensors. Based on the excellent catalytic activity and electron conduction ability of SnO_2_-rGO, the sensor achieved highly sensitive synchronous analysis of Cd^2+^, Pb^2+^, Cu^2+^, and Hg^2+^, and the detection limits were 11.4, 38.1, 14.4, and 55.9 nM, respectively. 

**Figure 7:**
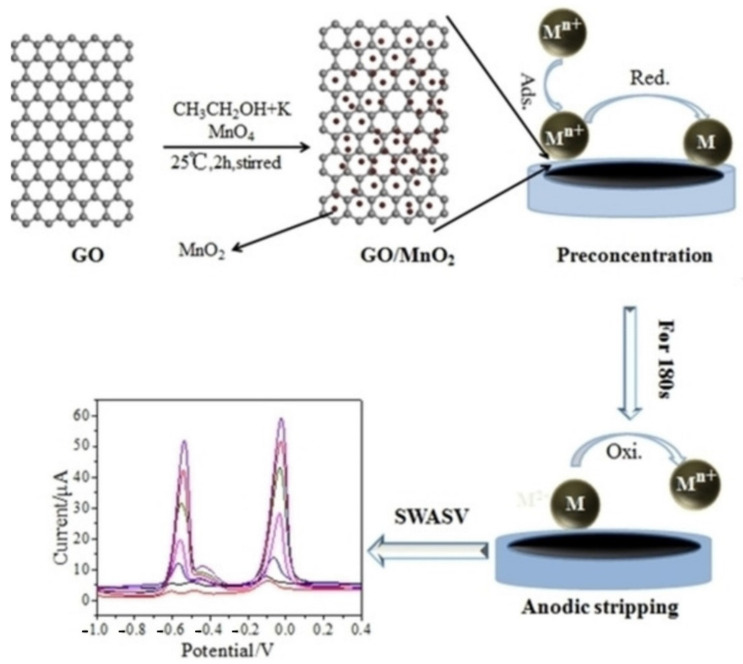
Schematic presentation on the fabrication of GO and MnO_2_-based electrochemical Cu^2+^ and Pb^2+^ sensors. Reprinted with permission from Ref. [188].

Sun et al. [188] prepared GO/MnO_2_ nanocomposites by a simple synthesis method and modified GCE for electrochemical detection of Cu^2+^ and Pb^2+^, as shown in Figure 7. It was found that MnO_2_ was uniformly attached to the layered structure of GO, forming more adsorption sites. Under the optimum conditions, the linear range of GO/MnO_2_/GCE for Cu^2+^ and Pb^2+^ was 0.05 to 1 μM, and the detection limits were 1.67 and 3.33 nM, respectively. Jiang et al. [189] prepared a polysulfide/graphene nanocomposite, which can be modified onto the carbon substrate using GO as the matrix and decorated with polysulfide groups, and used as an electrochemical sensing platform for highly sensitive and selective detection and analysis of Cd^2+^ and Pb^2+^. The detection ranges of Cd^2+^ and Pb^2+^ were 2.0–300 μM and 2.0–300 μM, respectively, and the detection limits were 0.67 μM and 0.17 μM, respectively.

#### 4.1.3. Detection of Antibiotics and Pesticides

Antibiotics and pesticide residues of animal and plant products in food safety have attracted more and more attention [190]. Long-term consumption of foods containing antibiotic residues may cause complications such as antibiotic resistance and allergic reactions. Therefore, it is very important to develop simple, sensitive and reliable methods to determine antibiotics and pesticide residues in food. Jiao et al. [191] prepared a novel composite membrane composed of carbon black and GO@Fe_3_O_4_. Based on this membrane, an electrochemical aptamer sensor with high sensitivity and selectivity was developed for the detection of a poisonous maidservant, and the detection limit reached 0.033 ng/mL. Zhou et al. [192] constructed an electrochemical sensor for the detection of pesticide residues using carboxylated graphene as the substrate and compounding SnO_2_ nanoparticles to form composites. Due to the synergistic effect between SnO_2_ nanoparticles and graphene, the composite had excellent electrical conductivity, electrocatalytic activity, and biocompatibility. The sensor could be used for sensitive detection of methyl parathion and carbofuran. The detection limits were 0.05 pM and 0.5 pM, respectively, and the linear detection ranges were 0.1 pM–0.1 nM, 0.1 nM–10 nM, 1 pM–0.1 nM and 0.1 nM–10 nM, respectively. 

In addition to graphene, other graphene-like 2DMs were utilized for the fabrication of electrochemical sensors for the detection of pesticide residues. For example, Kokulnathan et al. [193] used 2D BN and bismuth oxide to prepare electrochemical sensors for the detection of flutamide. Li et al. [194] used graphene embedded with AgNPs to form a multi-layer nanostructure and successfully analyzed and detected malachite green molecules in water. The lowest detection limit was 1 × 10^−11^ M. Malachite green is an additive mainly used in dyeing and aquaculture and is at risk of causing cancer in humans. Nasir et al. [195] prepared conductive 1T phase transition metal sulfide TMDs nanosheets (MoS_2_, MoSe_2_, WS_2_, WSe_2_) by tert-butyllithium stripping. Using 1T phase TMDs nanosheets (MoS_2_, MoSe_2_, WS_2_, WSe_2_) as platforms, indirect electrochemical detection of organophosphorus pesticide fenitrothion was carried out by enzyme inhibition. Compared with the sensors without modification of 1T phase TMDs nanosheets, the sensors modified with MoS_2_, MoSe_2_, WS_2,_ and WSe_2_ nanomaterials showed enhanced electrochemical response. Among them, 1T phase WS_2_ was better than all the other three TMD materials. The sensor system can detect fenitrothion pesticides in a wide concentration range of 1~1000 nM, with good linearity (r = 0.987), high sensitivity, and very low detection limit (2.86 nM). 

**Figure 8:**
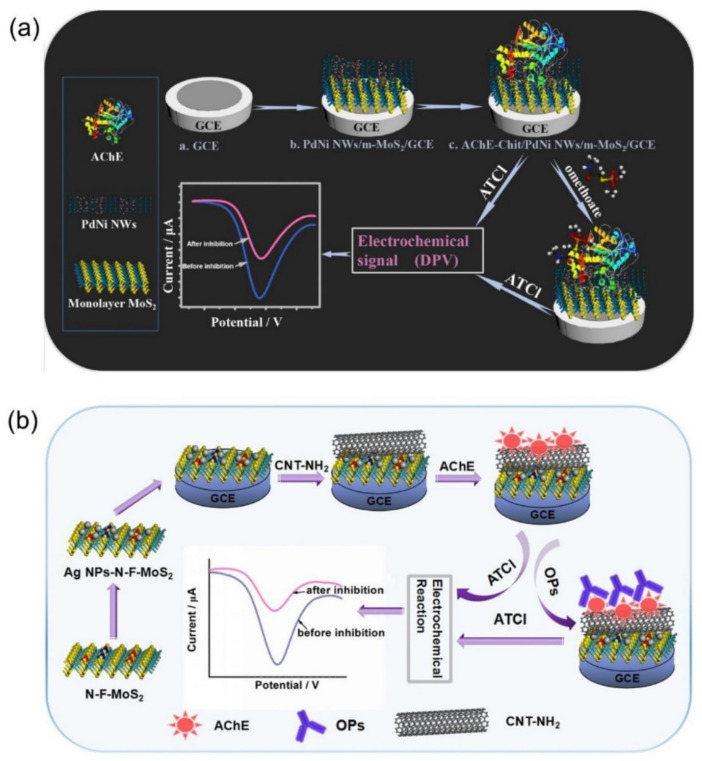
(**a**) Schematic illustration of the fabrication process of the electrochemical biosensor for omethoate assay [196]. (**b**) Schematic illustration of the fabrication process of the electrochemical biosensor for determination of monocrotophos and chlorpyrifos [197].

Song et al. [196] prepared a composite of ultra-thin bimetallic alloy nanowires (PdCu NW, PdCo NW, PdNi NW) and monolayer molybdenum disulfide nanosheets (m-MoS_2_) with 3D porous structure by one-step synthesis. By using this composite as electrode material, a novel sensor was constructed for electrochemical detection and analysis of omethoate, a typical highly toxic organophosphorus pesticide (Figure 8a). Under the optimal conditions, the linear range of the sensor for omethoate detection was 10^−13^ M~10^−7^ M, and the lowest detection limit reached 0.05 pM. In addition, Song et al. [197] prepared a few layers of N-F co-doped MoS_2_ nanosheets by hydrothermal method and then prepared AgNPs-N-F-MoS_2_ nanocomposites by a simple in situ growth method, which was used to construct electrochemical sensors for rapid detection of the most commonly used organophosphorus insecticides monocrotophos and chlorpyrifos, as shown in Figure 8b. The linear range for detection of monocrotophos was 10^−10^ ~10^−6^ mg/mL, and the detection limit was 0.05 pg/mL. Similarly, the linear range for chlorpyrifos was 5 × 10^−8^~10^−7^ mg/mL and 10^−7^~10^−4^ mg/mL, and the detection limit was 1 pg/mL. Jiang et al. [198] prepared a composite material of Ti_3_C_2_T_x_ by in situ reductions and used it to assemble an electrochemical sensor to detect pesticide residues. Through the synergistic effect between AgNPs and Ti_3_C_2_T_x_ nanoparticles, the sensor realized the hypersensitive detection of malathion with a detection limit of 3.27 × 10^−15^ M and a detection range of 10^−14^–10^−8^ M.

In addition, Peng et al. [199] prepared layered MoS_2_/graphene nanocomposites by solvothermal method and used them as substrates to construct electrochemical sensors for the detection of hydroquinone. It was found that the synergistic effect of nanomaterials makes the nanocomposites have good electrocatalytic performance for the electrochemical reaction of hydroquinone. Under the optimal conditions, the MoS_2_/graphene-modified electrode could be used for sensitive detection of hydroquinone in the range of 1–9 nM, with a detection limit of 0.3 nM. In addition, the fabricated electrochemical sensor exhibited good stability, selectivity, and reproducibility. In another case, Wu et al. [200] used hydrofluoric acid as raw material to peel off the original phase of Ti_3_AlC_2_ to obtain MXene-Ti_3_C_2_ 2DMs with electrical conductivity equivalent to that of multilayer graphene. Because Mxene-Ti_3_C_2_ has metal conductivity, biocompatibility, and good aqueous phase dispersion, the prepared Mxene-Ti_3_C_2_ was used as the matrix for immobilization of tyrosinase, and an electrochemical sensor was constructed to realize the ultra-sensitive and rapid detection of phenol. The linear range was 0.05–15.5 μM, the detection limit was 12 nM, and the sensitivity was 414.4 mA mol L^−1^.

### 4.2. 2DM-Based Electrochemical Sensors/Biosensors for Biomolecular Detection

Electrochemical sensors have realized the transformation from ion and gas electrodes to biological electrodes. At the same time, the vigorous development of biotechnology also provided great help to the development of electrochemical biosensors. Because the electrochemical biosensor has the advantages of both electrochemical and biological detection, it received extensive attention and played an important role in the field of sensors [201,202]. The nanomaterials suitable for the construction of electrochemical biosensors should have the following characteristics: (1) good electrical conductivity; (2) the surface area is large enough to provide a wider contact surface for the tested molecules; (3) good biocompatibility; (4) good biological stability and can still work normally after being preserved for a period of time; (5) low cost, simple, and environmentally friendly manufacturing process.

#### 4.2.1. Detection of Glucose, AA, UA, and Other Small Molecules

2DMs have a large specific surface area and can absorb a large number of substances to be measured, which is conducive to the increase in electron transfer rate, electrochemical reaction rate, and detection sensitivity [203]. Therefore, 2DMs have a good application prospect in the detection of small biological molecules. At present, it has been applied to the detection of H_2_O_2_, glucose, ascorbic acid (AA), uric acid (UA), dopamine (DA), tryptophan, and other small biological molecules [204,205,206]. 

**Figure 9:**
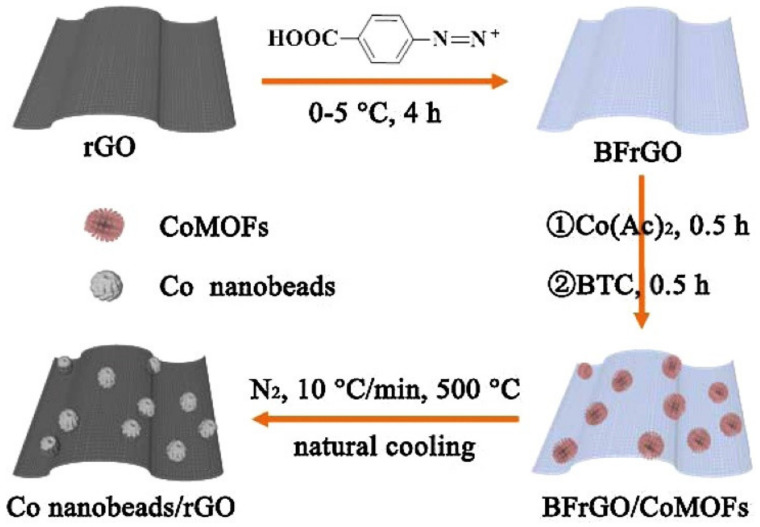
Schematic illustrating preparation process of 3D hierarchical Co nanobeads/rGO nanocomposites. Reprinted with permission from Ref. [208].

For example, Wu et al. [207] used the exfoliated MoS_2_ nanosheets to be reduced by electrochemical reduction in NaCl solution to produce reduced MoS_2_ (rMoS_2_). The resulting rMoS_2_ can detect glucose molecules by immobilization of GOx, and can selectively detect DA in the presence of UA and AA. Song et al. [208] firstly combined the benzoic acid group with reduced graphene oxide via a C-C bond to obtain benzoic acid-functionalized rGO (BFrGO). Then, BFrGO was immersed in a solution containing Co^2+^ and pyromellitic acid to form BFrGO/Co-MOFs step by step, and then BFrGO/Co-MOFs were decomposed by high-temperature calcination to obtain novel porous cobalt nanospheres/rGO nanocomposites, as shown in Figure 9. A novel electrochemical sensor for glucose detection was constructed based on porous cobalt nanospheres/rGO nanocomposites. Porous cobalt nanospheres/rGO nanocomposites have porous and interfacial layering, good electrical conductivity, and a large specific surface area, so the sensor has good detection performance for glucose, and the detection range was 5–1200 μM, the detection limit was 0.31 μM.

Huang et al. [23] modified copper nanoparticles (Cu NPs) onto MoS_2_ nanoflakes by chemical reduction. The composite sensor for the detection of glucose showed synergistic electrocatalytic oxidation activity with a sensitivity of 1055 μA mM^−1^ cm^−2^ and a linear range of more than 4 mmol L^−1^. Parlak et al. [209] constructed the MoS_2_/AuNPs interface on the surface of a glassy carbon electrode by electrostatic adsorption, which not only accelerated the surface electrocatalytic reaction but also showed excellent electrochemical performance, such as high current density, electron transfer rate, fast mass transfer rate and so on. The constructed MoS_2_/Au NPs/GOx sensor interface has a good linear range of 0.25~13.2 mmol L^−1^ in the detection of glucose, the detection limit was 0.042 μmol L^−1^, and the sensitivity was 13.80 μA μ M^−1^ cm^−2^. In addition, Kavitha et al. [210] prepared graphene-ZnO nanosheets for glucose detection by glucose sensor, and the sensitivity to glucose was improved by composite materials. Liu et al. [211] developed a sensor for the detection of glucose by using Cu_2_O nanocubes-modified graphene nanosheets as electrodes. The sensor has a linear response to glucose in the concentration range of 0.3~3.3 mM, with a detection limit of 3.3 mM, high selectivity, and a short response time. Compared with unloaded Cu_2_O nanocubes, graphene-coated Cu_2_O nanocubes have higher catalytic activity for glucose oxidation, higher sensitivity, and lower detection limit. Wang et al. [212] prepared an electrode that highly dispersed palladium nanoparticles on graphene for the detection of glucose by electrochemical sensors.

#### 4.2.2. Detection of H_2_O_2_ and Other Small Molecules Related to Diseases

Reactive oxygen species (ROS), which play an important role in cell metabolism, are markers of progressive neurodegenerative diseases such as Alzheimer’s disease, Parkinson’s disease, and cancer. H_2_O_2_ can spread freely through the cell membrane and is a typical representative of ROS in organisms [213,214]. Controlling the content of H_2_O_2_ at an appropriate level is very important for the intracellular signaling pathway of normal cells, so it is necessary to monitor the level of H_2_O_2_ in the biological environment, especially in the cellular environment. Wang et al. [215] synthesized Ti_3_C_2_ nanosheets and used them to immobilize hemoglobin (Hb) to construct a mediator-free H_2_O_2_ biosensor with high stability and low detection limit. The experimental results show that Ti_3_C_2_ nanosheets show excellent biocompatibility to hemoglobin and maintain good biological activity and stability of hemoglobin. The sensor promotes the electron transfer of hemoglobin and shows excellent H_2_O_2_ detection performance. The detection range was 0.1–260 μM, and the detection limit was 20 nM. Lorencova et al. [24] detected the H_2_O_2_ of the synthesized Ti_3_C_2_ nanosheets in the cathodic potential. The experimental results show that Ti_3_C_2_ is an excellent catalyst for H_2_O_2_ reduction, and the detection limit of the sensor was as low as 0.7 nM, which is comparable to that of the best reported H_2_O_2_ sensor (0.3 nM). Tian et al. [216] obtained ultra-thin *g*-C_3_N_4_ nanosheets by liquid phase stripping of *g*-C_3_N_4_ bulk materials and studied the electrochemical properties of the materials. It was found that *g*-C_3_N_4_ nanosheets also had good electrocatalytic activity for H_2_O_2_ reduction.

In addition, 2DMs have a layered structure and a large specific surface area, which enables this material to be used as a substrate for other nanomaterials, including precious metals, transition metals, carbon materials, and conductive polymers. The combination of this nanomaterial further improves the performance of its electrochemical sensor. Therefore, the modification of precious metal nanoparticles on the surface of 2DMs is an effective method to increase electrochemical signals, of which gold nanoparticles are the most commonly used because AuNPs have excellent electrical properties and biocompatibility [217,218,219]. For example, Au modified MoS_2_ complex can detect AA, DA, and UA at the same time [220]. In addition, the sensor has an electrocatalytic activity for the oxidation of bisphenol A, the linear range was 0.05–100 μM, and the detection limit was 5.0 × 10^−9^ M [221]. Zhang et al. [222] synthesized AuNPs-rGO through a one-step method. AuNPs can be uniformly loaded on flake graphene, and the size of AuNPs can meet the expected requirements (2–3 nm). The detection limit of AuNPs-rGO modified glassy carbon electrode for H_2_O_2_ was 0.45 mM, and the detection sensitivity was 283 μA mM^−1^ in the range of 10–130 μM hydrogen peroxide. 

**Figure 10:**
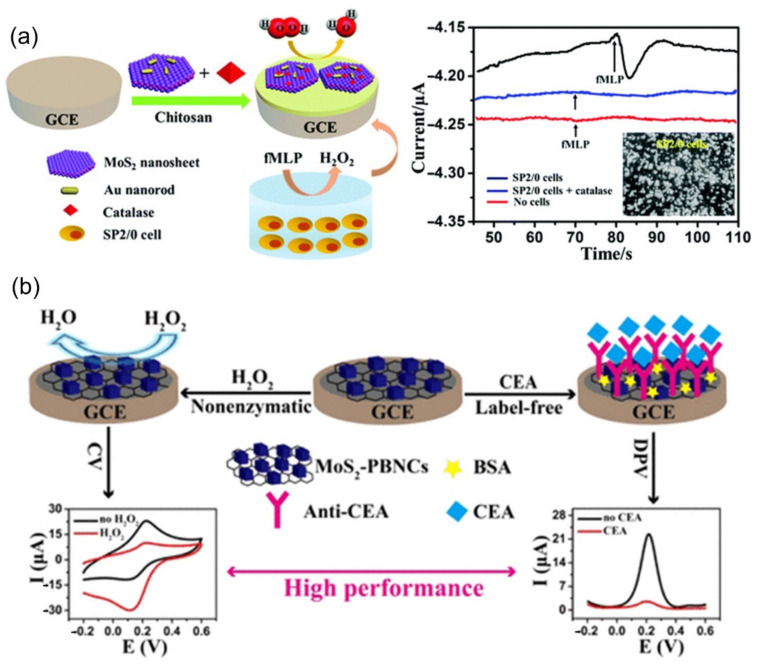
(**a**) Schematic of the catalase/MoS_2_–Au/chitosan modified GCE used for detecting H_2_O_2_ released from cells stimulated with fMLP (left) and Amperometric responses of the CAT/MoS_2_–Au/chitosan/GCE for the reduction in H_2_O_2_ released from about 8.0 × 10^7^ SP_2_/0 cells in 4 mL 1× PBS (pH 7.4) solution upon addition of 5 μM fMLP (right) [223]. (**b**) Illustration of MoS_2_-based electrochemical sensors for H_2_O_2_ (left) and CEA (right) Detection [224].

Shu et al. [223] constructed an electrochemical H_2_O_2_ biosensor based on MoS_2_ nanosheets–Au nanorods (Au NRs) complex to measure the release of H_2_O_2_ from living cells (as shown in Figure 10a). Firstly, the MoS_2_-Au NRs complex, which combines the advantages of MoS_2_ nanosheets and Au NRs, was prepared, and catalase (CAT) was immobilized on the surface of GCE. The unsaturated sulfur atoms at the edge of MoS_2_ nanowires can control the aggregation of nanoparticles. The large specific surface area of MoS_2_ nanosheets and the good biocompatibility of Au NRs enable the immobilized CAT to retain its natural structure and biological activity. In the real-time monitoring of H_2_O_2_ released from SP2/0 cells, N-formyl-methionyl-leucyl-phenylalanine (fMLP) was selected as a stimulant for the cell to generate H_2_O_2_. The 1 × PBS buffer solution without cells (red line in Figure 10a) and 1 × PBS buffer solution with cells and catalase (blue line) show no current change. While after the addition of fMLP, a significantly increased current was observed at the CAT/MoS_2_-Au/chitosan/GCE electrode, further suggesting that H_2_O_2_ was released from cells under the stimulation of fMLP. The results demonstrate that the constructed biosensor is sensitive and reliable for intracellular H_2_O_2_ detection. The biosensor revealed high sensitivity (187.4 mA M^−1^ cm^−2^), wide linear range (5.0 × 10^−7^ M~2.0 × 10^−4^ M), high selectivity and stability for H_2_O_2_ detection, and can be used to determine the trace concentration of H_2_O_2_ released by SP2/0 cells (detection limit of 0.1 μM). Su et al. [224] modified a multifunctional electrochemical sensor with MoS_2_-PBNCs nanocomposites (Figure 10b). The electrochemical sensor is used to detect the concentration of hydrogen peroxide and carcinoembryonic antigen, so it can be used in electrochemical catalysis and biomolecule detection.

**Figure 11:**
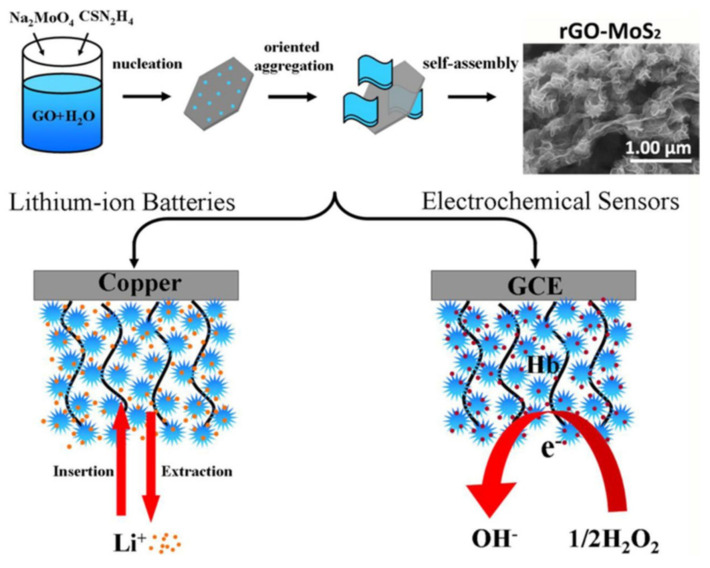
Schematic diagram of the applications of the MoS_2_-rGO hybrid material in Li-ion battery and electrochemical sensors [228].

In addition to metal nanoparticles, carbon materials, conductive polymers, and transition metals can also be used to modify nanosheets to improve their conductivity and surface area, thus improving electrochemical properties [225,226,227]. For example, Liu et al. [228] combined MoS_2_ with rGO hydrothermally to successfully synthesize MoS_2_-rGO composites, as shown in Figure 11. Then it was used to immobilize Hb, and a mediator-free biosensor was successfully constructed. The electrode showed an excellent electrochemical response to hydrogen peroxide with a wide detection range (0.1~250 μM), a high sensitivity of 346.6 μA mM^−1^ cm^−2^ and a detection limit of 25 nM. Wang et al. [145] successfully loaded TiO_2_ nanoparticles on Ti_3_C_2_ MXene materials to prepare TiO_2_-Ti_3_C_2_ nanocomposites with excellent properties and immobilized Hb on the surface of the composites to build a dielectric-free biosensor. The unique accordion-like structure of the composite accelerates the direct electron transfer rate of Hb, and the sensor showed excellent sensing performance in the detection and analysis of H_2_O_2_, with a sensitivity of 447.3 μA mmol L^−1^ cm^−2^, a linear range of 0.1~380 μM, and a very low detection limit of 14 nM.

#### 4.2.3. Detection of Medical Drugs, DNA, Protein, Antigen, and Others

The detection of other small molecules can be achieved by 2DM-based electrochemical sensors. For instance, Wang et al. [229] fabricated an electrochemical aptamer sensor based on AuNP-modified MoS_2_ nanosheets and β-cyclodextrin (MoS_2_-AuNPs-β-CD), which was developed for the hypersensitive detection of ochratoxin A (OTA). Using MoS_2_ as the dispersion template, MoS_2_-AuNPs nanocomposites with unique characteristics and functions were prepared by the in situ chemical reduction method. AuNPs promoted electron transfer, improved capture efficiency, and amplified the sensing signal. The constructed sensor realizes the detection of OTA with high sensitivity and selectivity. In another case, Jiang et al. [230] designed and prepared Ti_3_C_2_ MXene (ZnON/Ti_3_C_2_) heterostructure modified by ZnO quantum dots with high nitrogen doping level through simple heat treatment using glycine as an N precursor. By using it as an efficient electrochemical sensing platform, an electrochemical sensor sensitive and selective to chloramphenicol was constructed, which exhibited a wide linear range (0.1 ng/mL~100 ng/mL), low detection limit (0.019 ng/mL), and high stability. Gu et al. [231] prepared layered composites from graphene and *g*-C_3_N_4_ nanosheets and modified them on the surface of GCE. By using the properties of the composites to promote electron transfer and optimize redox current, a series of small biological molecules, including uric acid, norepinephrine, tryptophan, paracetamol, and rutin could be detected successfully. 

To make it more clear, Table 1 summarizes the performance of small molecule electrochemical sensors based on different 2DMs.

In addition, electrochemical sensors based on 2DMs and their composites can also be used in other biosensor fields, such as nucleic acid detection, protein detection, and so on. For example, Liu et al. [4] constructed a novel electrochemical immunosensor with a double-antibody sandwich structure based on rGO-Au NPs modified GCE, which was used to determine procalcitonin (PCT), a marker of septicemia. This method was successfully applied to the determination of PCT in blood samples. Yola et al. [242] constructed an electrochemical sensor based on GO-AuNPs to determine the content of tyrosine (Tyr) in milk. The detection limit could reach 0.15 nM. As a biomarker, Tyr is an important part of neurotransmitters, signal transduction systems, and hormones in the human brain [243]. It can prevent aging and senile dementia. It also has electrochemical activity and can be detected directly by electrochemical methods without other indirect processes such as derivatization. Wang et al. [104] proposed a sandwich structure immunosensor, which is a carcinoembryonic antigen (CEA) biosensor based on the MoS_2_-Au complex. The MoS_2_-Au complex is used to immobilize the first antibody (Ab1) of CEA, and the Ag nanoparticles are used to support the second antibody (Ab2) and GOx of CEA. When glucose exists in the system, the resulting H_2_O_2_ is catalyzed by the MoS_2_-Au complex, and the reduction peak can be detected. The immunosensor had a linear relationship in the range of 1–50 ng mL^−1^, and its detection limit could reach 0.27 pg mL^−1^. Huang et al. [244] also reported an electrochemical immunosensor using graphene nanocomposites coated with Ag/AuNPs to modify the bare working electrode for hypersensitive detection of carcinoembryonic antigen.

## 5. Conclusions and Future Perspectives

Based on the above introduction and discussion, it can be concluded that 2DM-based electrochemical sensors have significant advantages in substance detection, such as convenience and speed, simple operation, low cost, and short time consumption. They have excellent performance in food safety, environmental monitoring, biological science, pharmaceutical industry, and other fields. As a new research field in recent years, the development and application of electrochemical sensors based on 2DMs provide a new research topic for the detection of pesticide residues, heavy metal ion detection, and biological small molecule detection. This review mainly introduces the preparation methods, structures, and properties of 2DMs and their applications in various electrochemical detection. Through the design of functional nanomaterials and the construction of sensing electrodes, the test performance of electrochemical sensors is improved.

However, there are still some problems in the use of 2DM-modified electrodes for electrochemical sensor applications. (1) The preparation process of some nanomaterials is cumbersome, with poor repeatability and low service life, which seriously reduces the reliability of the test results. (2) Most nano-sensing materials and electrodes are bound by physical interaction, and the weak binding strength affects the stability of the sensor test. In order to solve the above problems, the following solutions can be taken. First, the design and synthesis of new nanomaterials with excellent electrochemical performance and stable structure are needed, which can significantly improve the reproducibility and reliability of sensing tests. Second, novel fabrication techniques for modifying electrodes should be explored and developed. By continuously improving the bonding mechanism between nanomaterials and modified electrodes, the bonding strength of nanomaterials on the electrode surface can be improved to enhance the stability and easy detachment of nanomaterial modifications on the electrode, thereby improving the stability of electrochemical detection.

## Figures and Tables

**Table 1:** The performances of small molecule electrochemical sensors based on different 2DMs.

Sensor Materials	Analyte	Range of Detection	Detection Limit	Refs.
MoS_2_	H_2_O_2_	5.0–100 nM	2.5 nM	[232]
MoS_2_-graphene-horseradish peroxidase	H_2_O_2_	0.2 µM–1.103 mM	0.049 µM	[233]
MoS_2_-PtW	H_2_O_2_	1–0.2 mM	5 nM	[234]
MoS_2_-Cu nanoflower	H_2_O_2_/glucose	0.04–1.88 µM/1–20 µM	0.021 µM/0.32 µM	[235]
MoS_2_-Ni NP	Glucose	0–4 mM	0.31 µM	[102]
MoS_2_-Au NP-glucose oxidase	Glucose	10–300 µM	2.8 µM	[236]
MoS_2_-PANI-Au NP	DA	1–500 µM	0.1 µM	[237]
MoS_2_-Au NP	DA	0.1–200 µM	80 nM	[238]
rGO	UA	0.02–0.49 mM	3.45 µM	[239]
MoS_2_-Ag	Tryptophan	0.5–120 µM	0.05 µM	[240]
MoS_2_- self-doped polyaniline	Chloramphenicol	0.1–1000 µM	6.5 × 10^−8^ M	[241]

## Data Availability

Not applicable.
